# Supplementary material for: Relationship between interoceptive sensibility and somatoform disorders in adults with autism spectrum traits. The mediating role of alexithymia and emotional dysregulation
Source: PLoS One. 2021 Aug 24;16(8):e0255460. doi: 10.1371/journal.pone.0255460 (PMC8384168; doi:10.1371/journal.pone.0255460)
Supplement: Attachment — Submitted filename: review somatoform dissociation.docx [file pone.0255460.s002.docx]

Review

This research aims to address an interesting question regarding the relationship between alexithymia, interoception and somatoform dissociation. However, it will need major revisions before it is ready for publication. The structure and clarity of the manuscript needs to improve a lot. I would like the authors to use paragraphs more. The clarity can further improve by improving grammar, be more precise, using clearer definitions, pointing out where research is contradicting or supporting of premises that the authors base their research question and hypotheses on. At the moment the introduction seems to be a list of descriptions of previous research, without much structure, and without a direction, i.e. how this research contributes to the research question and the justification of the hypotheses.

Methodological: - It is not clear if the participants in the autistic group actually have a diagnosis of ASC. If the participants haven’t got a formal diagnosis, this should be made clear. The groups should be based on diagnosis status, not AQ score, if the authors want to call one of the groups the ‘clinical group’ or ‘ASC group’. Participants without a diagnosis, even if they have a high AQ score, should be excluded from this group. Equally, participants with a diagnosis but with a low AQ score should not be included in the control group. If none of the participants have a diagnosis of autism, any suggestion that the high AQ group is the autistic group should be removed. If participants with diagnosed ASC form a group, their diagnosis should ideally be confirmed by a better diagnostic tool than the AQ, such as the ADOS for researchers.

In detail:

Abstract: First sentence unclear -improve grammar: Which concepts are causally linked? What is ordered alexithymia? Or, what does ‘causally linked and ordered’ mean? Express yourself more precisely and accurately: The study hasn’t used a population with ASC. The AQ doesn’t ‘measure autism’. Why does the abstract refer to models (plural) when only one statistical result is mentioned (don’t mention the actual statistics in the abstract).

The last sentence of the abstract doesn’t make sense, because of poor grammar.

Introduction:

1. Paragraphs are needed (in addition to the existing subheadings).
2. The authors need to explain all relevant concepts of the research question early on. This includes not only autism (which has been done to some extent), but also interoception, alexithymia, emotional dysregulation and somatoform dissociation, and how these concepts are measured. Evidence that suggests a relationship (mediated or not) between interoception and dissociation should be presented before introducing the proposed mediating factors (with justifying evidence – why these factors?). This should be followed by a justification why this is of interest in the autistic population in particular.

Page 3

3. The description ‘in terms of continuous traits’ is incorrect.
4. It doesn’t logically follow (‘thus’) that it is characterised by the co-occurrence of other psychological disorders.
5. Citation needed for the association between looking less at faces and difficulties recognizing emotions, facial expressions, the reading of minds. Avoid referring to causality (‘as a consequence’, ‘another consequence’) unless there is evidence for this (which should be cited).
6. Uljarevic’s work undermines the authors’ previous statements. Be more clear/precise about your claims about emotion recognition and how Uljarevic’s meta-analysis fits with this.
7. The authors need to explain what alexithymia and interoception is.
8. It is unclear why this focus on emotion recognition is relevant to your research question/hypotheses. Make this clear early on.
9. Definition of IA unclear or incorrect: what is meant by ‘together with external stimuli’? Typo: missing out the o in interoceptive processes.
10. Citation needed for the claim that ‘the concept’ (?) of being aware/unaware has an influence on many behaviours that a person displays automatically (as opposed to unautomatic?). Unclear.
11. Is there difference between internal body sensations (interoceptive accuracy) and internal sensations (interoceptive sensibility)? Be consistent in how things are described/defined.
12. It is unclear if or how Rogers’ study applies to interoception, as this appears to be about exteroceptive stimuli. Explain why this is mentioned.
13. ‘catastrophic effects’? Avoid strong language like this unless there is evidence (which you’d need give examples of).
14. The authors switch from a phenomenological description of sensory processing to a neurological description (Pellicano and Burr) without indicating this. There currently isn’t hard evidence that a predictive coding account of autism can be directly equated with predictions on an empirical, let alone conscious, level. This is unclear in the way Pellicano’s research is described in this section. Spelling error: contend. And again, it is unclear how this research is relevant to the research question/hypotheses.
15. What ‘positive idea’? What is ‘this’? Why is it positive (as opposed to negative?)? What is meant by ‘pursuing a relation to emotion-related experiences’?

Page 5.

16. Garfinkel’s study builds on Pellicano and Burr’s proposition. So, if relevant, explain better how their findings fit with a predictive coding account of autism – i.e. explain what a prediction error is (are they right to call their calculation a prediction error?). But more importantly, make clear what the relevance of the predictive coding account of autism is to the research question/hypotheses. If it isn’t relevant, consider leaving it all out.
17. What is meant by ‘an origin or emotion deficits and affective symptoms in ASC **at the interface between body and mind**? What is ‘expectancy-driven interpretation of interoceptive information’? Only someone who already understands the predictive coding account of sensory processing in autism would perhaps be able to guess the meaning of this sentence. For everybody else, it is just plainly unclear what is meant.
18. ‘Atypical forms of interoceptive awareness’: Are there multiple forms of atypical interoceptive awareness in autism and alexithymia? Expand and explain. This does seem relevant to your research question.
19. The switch to emotion regulation is too sudden. Nothing has been mentioned about emotion regulation in autism yet, but ‘disorders in emotion regulation’ is mentioned as if it is relevant. Do the authors imply that ASC is a disorder of emotion regulation?
20. What does ‘a cluster of cognitive and affective features **in terms of** the alexithymia construct’ mean? If you mean to describe alexithymia, please make clear that you are describing alexithymia. Here it seems the authors are describing emotion regulation, badly, unclearly and incorrectly.

Page 6.

21. What is ‘interoceptive touch’? How/why is this relevant to the research question?
22. Why do the authors bring up interoceptive touch after a section about alexithymia? Why are the sentences about ‘touch atypicalities’ followed by a section about affective and non-affective interoceptive states? Is there a relation?
23. Is interoception perceiving an interoceptive state? What are affective and non-affective interoceptive states? What is the difference between an ‘affective interoceptive state’ and an emotion? Should this not have been explained earlier, when interoception was explained? Is this distinction relevant to the research question? Explain.
24. The last sentences of page 6 is a repetition of the one before.

Page 7.

25. It doesn’t follow that if alexithymia is characterized by a general failure of interoception, that ‘accordingly’ people with ASC and alexithymia have higher interoceptive sensitivity and an increased ANS activation, ‘which *in turn* **doesn’t** correlate with interoceptive accuracy’. This doesn’t make sense. Explain better. Be consistent in the use of the terminology: interoceptive sensitivity, interoceptive sensibility and interoceptive accuracy. Furthermore, it should be pointed out that research into interoceptive processing in autism is not conclusive; studies have found lower interoceptive accuracy, and both lower and higher interoceptive sensibility, in autistic individuals. The Garfinkel study is far from the only study that investigated this. The authors should read and cite, where relevant, the studies of Fiene (2015), Mul et al (2018), Nicholson et al (2018,2019), Palser et al (2018), Shah et al (2016; 2018)
26. Explain what the characteristics of dissociation are and how this is relevant to ASC. Why do the authors start with dissociation in childhood trauma? Because the authors don’t explain if or how or why dissociation occurs in individuals with ASC it appears they imply that autism can be compared with a trauma, which is problematic. It also appears the authors suggest that disorders develop as a ineffective resolution of trauma (‘do not resolve the trauma’). Unclear.
27. What is ‘psychological dissociation’ and ‘somatoform dissociation’? Symptoms of either should be explained much earlier, and in a concrete way. Not: ‘phenomena that are manifestations of a lack of integration of somatoform functions, reactions and experiences’ (page 8). It is very unclear what this means in real life.

Page 8

28. What are ‘animal defensive reactions’?
29. The authors’ “essential assumption” that alexithymia is due to a deficit in the cognitive processing of mental representations is NOT what was noted above. In fact, the impression is that the authors went out of their way to explain that alexithymia was due to a general failure of interoceptive processing. Please be clear and consistent.
30. More inconsistency: It appears that the authors think that the limited capacity to use cognitive mechanisms to understand and regulate their emotions (i.e. their alexithymia?) causes the amplification and misinterpretation of bodily signals, when a whole section above was written to persuade the reader of the opposite causal relationship.
31. Descriptions of dissociation and emotional dysregulation, and their relationship to autism, needs to be covered much earlier in the introduction. It needs to be explained why this needs to be investigated in autism.
32. It needs to be made clear that the associations described at the bottom of page 8 refer to subscales of the TAS-20, but I wonder how relevant it is to know that the relationship is less strong for externally oriented thinking.
33. Could the authors make the relevance of somatosensory amplification to somatosensory dissociation more clear? The research question is about somatosensory dissociation, after all. It is unclear why dissociation is the concept of interest in this study, and not somatosensory amplification.

Page 9

34. Please be clear and consistent on terminology: What is somatization? What are somatoform disorders? What are somatic disorders? Is there a difference between them and between the medical disorders mentioned? How are these disorders relevant to the concept supposedly under investigation: somatoform dissociation?

Page 10

35. The authors have not justified their research question and hypotheses regarding somatoform dissociation very well. It is unclear what somatoform dissociation actually is, why it is of interest in the autistic population, how it can be measured, and why emotion dysregulation is also a factor of interest, and why they propose a mediating role to alexithymia and emotion dysregulation (not emotion regulation?) in the relationship between interoception and somatoform dissociation.
36. The authors are not measuring ANS reactivity, so this should not feature in the hypothesis.
37. The description of the methods under 2) ‘(a battery of …’ is too vague and doesn’t constitute a justification. In the introduction, the authors have not presented a clear and coherent summary of ‘theoretical findings’ (?) that justify the research question, nor the hypothesised model. E.g. why a ‘parallel effect’? No details or explanation have been given.

Methods

Page 11

38. Please clarify whether participants in the ASC group have a formal diagnosis. If not, I think it would be problematic to call a group with high autistic traits the ASC group. If the participants haven’t got a diagnosis, this should be made clear and not be confused by a misnomer. If participants with ASC are included, their diagnosis should ideally be confirmed by a better diagnostic tool than the AQ, such as the ADOS. Have participants without a diagnosis but with high autistic traits been excluded from the non-ASC group (because they may have undiagnosed ASC and could be a confound)?
39. Inconsistent: the participant group is referred to as ASC as well as ASD. If the group doesn’t actually consist of individuals with ASC, it shouldn’t be referred to as such. High autistic traits vs. low autistic traits would be more appropriate than ASC vs control.
40. Explain the cut off point of the AQ.
41. It is not made clear which of the questionnaires measures interoception, and what type of interoceptive processing (accuracy, sensitivity, sensibility).
42. From the description of the questionnaires, it appears that there is some overlap between the DERS and the TAS-20. How are they different? Do they correlate? How meaningful are the findings if there is multicollinearity? In the mediation model, when assessing the relationship between the TAS-20 and somatoform dissociation, has the DERS been controlled for? And similarly, when assessing the relationship between DERS and somatoform dissociation, has TAS-20 been controlled for?

Results

Page 14

43. “A single factor analysis of variance was carried out in an intergroup schema” is a strange way to describe the analysis done. Please make clearer what the independent and dependent variables are. With multiple dependent variables, isn’t a manova more appropriate? It doesn’t seem any post-hoc analyses were done after the (m)anova. In the table, the post-hoc statistics should be reported.
44. In the description of the correlations, it is not always made clear which variables were correlated. Most of the time only one variable is mentioned, and it is unclear what other variable the correlation refers to.

Page 15

45. It is unclear why the authors have chosen to do a moderation analysis with high/low AQ as the moderator (as suggested by the graph) and not the two participant groups. In the graph, what does AQ yes AQ no mean? Surely all participants have an AQ result? Please use a more appropriate description. The description of the moderation analysis results is grammatically poorly written and is repetitive. The one result is described multiple times as if each description is a new finding.

Page 16

46. It is unclear how the ‘obtained results’ of the moderation analysis ‘form the basis’ for the mediation analyses. Also, that authors state that it confirms the relationship in the ‘examined group’ (singular), not groups (plural). This is confusing, as it is unclear which group the authors refer to, and also, the moderation was confirmed for both groups.
47. Please don’t use the word ‘proven’

Page 17

48. Here the authors specifically mention that the analysis shows (not: ‘achieved’) a partial mediation. This was however also the case with the two earlier mediation analyses.
49. In the summary of the mediation analyses results, the authors mention only the ASC group. Were the mediation analyses carried out on data from the ASC group only, not all participants? This is unclear, and if so, why? Why haven’t the authors carried out a moderated mediation analysis, which is possible in PROCESS? This would show the effect of the moderation of autism status on the mediated relationship between interoception and somatoform dissociation.

Discussion

Page 18

50. Please state the results in terms of the research question and hypotheses – why and how were the RQ and hypotheses formulated – ‘to verify a model’ is not informative. Again, sentences are poorly constructed, which impedes clarity. Please be consistent in the use of acronyms (what is DIF? Why do the authors write out somatoform dissociation in full, but use an acronym for SA, which wasn’t measured in the study, and the reader may have forgotten what it stands for.
51. The authors should make clear when they are speculating about mechanisms that may explain the findings, such as SA. Some statements are too strong and suggest causality when they can’t be sure of that.
52. The Discussion suffers from the same problem as the introduction: a list of descriptions of previous research, of which the relevance (to the authors’ findings this time – in the introduction it’s the relevance to the hypotheses and research question) is not made clear; supporting and opposing research findings are not clearly pointed out (everything is treated as supporting, even when it’s not); definitions are not clear – somatoform dissociation is equated with physical problems, physical illness, somatic amplification etc. It doesn’t address the question why these findings are worth knowing – how will this research help people with autism or what is its contribution to knowledge specifically?
