# Supplementary material for: Relationship between interoceptive sensibility and somatoform disorders in adults with autism spectrum traits. The mediating role of alexithymia and emotional dysregulation
Source: PLoS One. 2021 Aug 24;16(8):e0255460. doi: 10.1371/journal.pone.0255460 (PMC8384168; doi:10.1371/journal.pone.0255460)
Supplement: Attachment — Submitted filename: Response to Reviewers.docx [file pone.0255460.s003.docx]

Dear Reviewers,

We would like to express our gratitude to the Reviewers for valuable remarks regarding the manuscript and feedback on how to improve and enhance the content of the manuscript.

We really appreciate your remarks, therefore we have modified the text according to the suggestions that we received. Due to the fact that the text has been modified in approximately 70-80 per cent, we have decided to mark modifications in blue colour in "Revised Article with Changes Highlighted" file, and we upload new tables and figures.

We hope that the changes in the text will meet your expectations, and we will look forward to receiving your response.

Yours sincerely,

Review

This research aims to address an interesting question regarding the relationship between alexithymia, interoception and somatoform dissociation. However, it will need major revisions before it is ready for publication. The structure and clarity of the manuscript needs to improve a lot. I would like the authors to use paragraphs more. The clarity can further improve by improving grammar, be more precise, using clearer definitions, pointing out where research is contradicting or supporting of premises that the authors base their research question and hypotheses on. At the moment the introduction seems to be a list of descriptions of previous research, without much structure, and without a direction, i.e. how this research contributes to the research question and the justification of the hypotheses.

Methodological: - It is not clear if the participants in the autistic group actually have a diagnosis of ASC. If the participants haven’t got a formal diagnosis, this should be made clear. The groups should be based on diagnosis status, not AQ score, if the authors want to call one of the groups the ‘clinical group’ or ‘ASC group’. Participants without a diagnosis, even if they have a high AQ score, should be excluded from this group. Equally, participants with a diagnosis but with a low AQ score should not be included in the control group. If none of the participants have a diagnosis of autism, any suggestion that the high AQ group is the autistic group should be removed. If participants with diagnosed ASC form a group, their diagnosis should ideally be confirmed by a better diagnostic tool than the AQ, such as the ADOS for researchers.

The title has been changed “Relationship between Interoceptive Sensibility and Somatoform Disorder in Adults with Autism Spectrum Traits. The Mediating Role of Alexithymia and Emotional Dysregulation”

It is extremely difficult to refer item by item to the comments below due to the fact that the article has been revised radically in accordance with the below remarks. At this point, it is the content of the amended article that requires analysis.

However, we will try to answer and refer to the below remarks, comments, and questions.

In detail:

Abstract: First sentence unclear -improve grammar: Which concepts are causally linked? What is ordered alexithymia? Or, what does ‘causally linked and ordered’ mean? Express yourself more precisely and accurately: The study hasn’t used a population with ASC. The AQ doesn’t ‘measure autism’. Why does the abstract refer to models (plural) when only one statistical result is mentioned (don’t mention the actual statistics in the abstract).

The last sentence of the abstract doesn’t make sense, because of poor grammar.

The Abstract has been fully changed in accordance with the Reviewer’s comments. The clinical group and the control group have also been described in detail.

Introduction:

1. Paragraphs are needed (in addition to the existing subheadings).
2. The authors need to explain all relevant concepts of the research question early on. This includes not only autism (which has been done to some extent), but also interoception, alexithymia, emotional dysregulation and somatoform dissociation, and how these concepts are measured. Evidence that suggests a relationship (mediated or not) between interoception and dissociation should be presented before introducing the proposed mediating factors (with justifying evidence – why these factors?). This should be followed by a justification why this is of interest in the autistic population in particular.

In line with the comments, the structure of the entire article has been modified. At the beginning, there is an Introduction, where the relationships between interoception and somatic disorders in the context of alexithymia and dysregulation are presented. Further, there are paragraphs on the characteristics of persons with ASD in respect of investigated aspects and conditions.

Page 3

3. The description ‘in terms of continuous traits’ is incorrect.

This point has been changed and described appropriately.

4. It doesn’t logically follow (‘thus’) that it is characterised by the co-occurrence of other psychological disorders.

The sentence has been changed.

5. Citation needed for the association between looking less at faces and difficulties recognizing emotions, facial expressions, the reading of minds. Avoid referring to causality (‘as a consequence’, ‘another consequence’) unless there is evidence for this (which should be cited).

This fragment has been supplemented appropriately, and the wording has been changed.

6. Uljarevic’s work undermines the authors’ previous statements. Be more clear/precise about your claims about emotion recognition and how Uljarevic’s meta-analysis fits with this.

Studies have been added before the meta-analysis showing the link between socio-emotional disorders with alexithymia, and not with ASD.

7. The authors need to explain what alexithymia and interoception is.

Definitions of alexithymia and interoception have been added.

8. It is unclear why this focus on emotion recognition is relevant to your research question/hypotheses. Make this clear early on.

The Introduction presents the results of studies which indicate the relationship between alexithymia and emotion dysregulation in the context of somatic consequences.

9. Definition of IA unclear or incorrect: what is meant by ‘together with external stimuli’? Typo: missing out the o in interoceptive processes.

Details concerning interoceptive awareness have been added.

10. Citation needed for the claim that ‘the concept’ (?) of being aware/unaware has an influence on many behaviours that a person displays automatically (as opposed to unautomatic?). Unclear.

The paragraph has been extended and changed.

11. Is there difference between internal body sensations (interoceptive accuracy) and internal sensations (interoceptive sensibility)? Be consistent in how things are described/defined.

We hope that in the new version of the article the definitions have been made more specific.

12. It is unclear if or how Rogers’ study applies to interoception, as this appears to be about exteroceptive stimuli. Explain why this is mentioned.

The results of studies have been presented indicating the role of excessive interoceptive sensibility in the context of external stimuli interpretation.

13. ‘catastrophic effects’? Avoid strong language like this unless there is evidence (which you’d need give examples of).

The term has been supplemented. An example of such meaning has been provided.

14. The authors switch from a phenomenological description of sensory processing to a neurological description (Pellicano and Burr) without indicating this. There currently isn’t hard evidence that a predictive coding account of autism can be directly equated with predictions on an empirical, let alone conscious, level. This is unclear in the way Pellicano’s research is described in this section. Spelling error: contend. And again, it is unclear how this research is relevant to the research question/hypotheses.

The text has been supplemented with reasoning for the presented study results.

15. What ‘positive idea’? What is ‘this’? Why is it positive (as opposed to negative?)? What is meant by ‘pursuing a relation to emotion-related experiences’?

The fragment has been changed.

Page 5.

16. Garfinkel’s study builds on Pellicano and Burr’s proposition. So, if relevant, explain better how their findings fit with a predictive coding account of autism – i.e. explain what a prediction error is (are they right to call their calculation a prediction error?). But more importantly, make clear what the relevance of the predictive coding account of autism is to the research question/hypotheses. If it isn’t relevant, consider leaving it all out.

In the revised part of the article the purpose of disregarding the context, and focusing on details in assigning wrong interpretations to signals from the body have been described.

17. What is meant by ‘an origin or emotion deficits and affective symptoms in ASC **at the interface between body and mind**? What is ‘expectancy-driven interpretation of interoceptive information’? Only someone who already understands the predictive coding account of sensory processing in autism would perhaps be able to guess the meaning of this sentence. For everybody else, it is just plainly unclear what is meant.

The part has been described in more detail.

18. ‘Atypical forms of interoceptive awareness’: Are there multiple forms of atypical interoceptive awareness in autism and alexithymia? Expand and explain. This does seem relevant to your research question.

The relationship between alexithymia and interoceptive awareness in the context of interoceptive sensibility has been discussed.

19. The switch to emotion regulation is too sudden. Nothing has been mentioned about emotion regulation in autism yet, but ‘disorders in emotion regulation’ is mentioned as if it is relevant. Do the authors imply that ASC is a disorder of emotion regulation?

This comment has been taken into account in the Introduction. The relationships between alexithymia, emotional dysregulation and somatoform disorders have been analysed.

20. What does ‘a cluster of cognitive and affective features **in terms of** the alexithymia construct’ mean? If you mean to describe alexithymia, please make clear that you are describing alexithymia. Here it seems the authors are describing emotion regulation, badly, unclearly and incorrectly.

The definition of alexithymia has been introduced and discussed in the Introduction.

Page 6.

21. What is ‘interoceptive touch’? How/why is this relevant to the research question?

Atypical interoceptive sensibility in people with ASD may also take the form of decreased or increased sensation thresholds, in this case the modality related to touch.

22. Why do the authors bring up interoceptive touch after a section about alexithymia? Why are the sentences about ‘touch atypicalities’ followed by a section about affective and non-affective interoceptive states? Is there a relation?

Alexithymia, as shown by the attachment theory, arises on the basis of non-secure attachment, especially anxiety-avoidant and disorganized, and relates to emotional neglect, which, among others, includes satisfying the need for direct tactile contact between a child and an attachment figure.

23. Is interoception perceiving an interoceptive state? What are affective and non-affective interoceptive states? What is the difference between an ‘affective interoceptive state’ and an emotion? Should this not have been explained earlier, when interoception was explained? Is this distinction relevant to the research question? Explain.

The issues have been supplemented in the Introduction.

24. The last sentences of page 6 is a repetition of the one before.

The sentence has been changed.

Page 7.

25. It doesn’t follow that if alexithymia is characterized by a general failure of interoception, that ‘accordingly’ people with ASC and alexithymia have higher interoceptive sensitivity and an increased ANS activation, ‘which *in turn* **doesn’t** correlate with interoceptive accuracy’. This doesn’t make sense. Explain better. Be consistent in the use of the terminology: interoceptive sensitivity, interoceptive sensibility and interoceptive accuracy. Furthermore, it should be pointed out that research into interoceptive processing in autism is not conclusive; studies have found lower interoceptive accuracy, and both lower and higher interoceptive sensibility, in autistic individuals. The Garfinkel study is far from the only study that investigated this. The authors should read and cite, where relevant, the studies of Fiene (2015), Mul et al (2018), Nicholson et al (2018,2019), Palser et al (2018), Shah et al (2016; 2018)

The fragment has been modified, supplemented, and it has been moved to the Introduction.

26. Explain what the characteristics of dissociation are and how this is relevant to ASC. Why do the authors start with dissociation in childhood trauma? Because the authors don’t explain if or how or why dissociation occurs in individuals with ASC it appears they imply that autism can be compared with a trauma, which is problematic. It also appears the authors suggest that disorders develop as a ineffective resolution of trauma (‘do not resolve the trauma’). Unclear.

In the part about ASD, studies have been quoted regarding non-secure attachment in the group of persons with ASD disorders and relational trauma, as well as the structure of "Dissociation, alexithymia - regulation of emotions and somatization" section has been changed.

27. What is ‘psychological dissociation’ and ‘somatoform dissociation’? Symptoms of either should be explained much earlier, and in a concrete way. Not: ‘phenomena that are manifestations of a lack of integration of somatoform functions, reactions and experiences’ (page 8). It is very unclear what this means in real life.

The terminology information has been entered in the Introduction, and supplemented in the “Dissociation, alexithymia - regulation of emotions and somatization" paragraph.

Page 8

28. What are ‘animal defensive reactions’?

This has been changed.

29. The authors’ “essential assumption” that alexithymia is due to a deficit in the cognitive processing of mental representations is NOT what was noted above. In fact, the impression is that the authors went out of their way to explain that alexithymia was due to a general failure of interoceptive processing. Please be clear and consistent.

We hope that the revised structure of the article and the theoretical Introduction show the nature of relationship between interocpetive sensibility and cognitive deficits in the ability to accurately recognize signals from the body as emotion correlates, and to verbalize emotions (alexithymia).

30. More inconsistency: It appears that the authors think that the limited capacity to use cognitive mechanisms to understand and regulate their emotions (i.e. their alexithymia?) causes the amplification and misinterpretation of bodily signals, when a whole section above was written to persuade the reader of the opposite causal relationship.

It is difficult for us to refer to this point at the moment, due to the changed structure of the article. It is important that alexithymia, which was initially shown, arises on the basis of disturbed attachment relations, as a result of which the child learns incorrect relations between the identification and verbalization of emotions. Cognitive distortions reversely cause the disturbed attribution of somatic states, favouring the development of interoceptive sensibility.

31. Descriptions of dissociation and emotional dysregulation, and their relationship to autism, needs to be covered much earlier in the introduction. It needs to be explained why this needs to be investigated in autism.

This has been changed accordingly.

32. It needs to be made clear that the associations described at the bottom of page 8 refer to subscales of the TAS-20, but I wonder how relevant it is to know that the relationship is less strong for externally oriented thinking.

It is important in terms of understanding the role of individual factors that comprise alexithymia syndrome in the etiopathogenesis of disorders.

33. Could the authors make the relevance of somatosensory amplification to somatosensory dissociation more clear? The research question is about somatosensory dissociation, after all. It is unclear why dissociation is the concept of interest in this study, and not somatosensory amplification.

More details have been added in the Introduction.

Page 9

34. Please be clear and consistent on terminology: What is somatization? What are somatoform disorders? What are somatic disorders? Is there a difference between them and between the medical disorders mentioned? How are these disorders relevant to the concept supposedly under investigation: somatoform dissociation?

The comments have been taken into account in the new text.

Page 10

35. The authors have not justified their research question and hypotheses regarding somatoform dissociation very well. It is unclear what somatoform dissociation actually is, why it is of interest in the autistic population, how it can be measured, and why emotion dysregulation is also a factor of interest, and why they propose a mediating role to alexithymia and emotion dysregulation (not emotion regulation?) in the relationship between interoception and somatoform dissociation.

This comment was fully taken into account and it is reflected in the new version of the article.

36. The authors are not measuring ANS reactivity, so this should not feature in the hypothesis.

The relevant corrections have been made.

37. The description of the methods under 2) ‘(a battery of …’ is too vague and doesn’t constitute a justification. In the introduction, the authors have not presented a clear and coherent summary of ‘theoretical findings’ (?) that justify the research question, nor the hypothesised model. E.g. why a ‘parallel effect’? No details or explanation have been given.

The structure of study results analysis has been changed according to the Reviewers’ comments, as well as other statistical analyses have been added.

Methods

Page 11

38. Please clarify whether participants in the ASC group have a formal diagnosis. If not, I think it would be problematic to call a group with high autistic traits the ASC group. If the participants haven’t got a diagnosis, this should be made clear and not be confused by a misnomer. If participants with ASC are included, their diagnosis should ideally be confirmed by a better diagnostic tool than the AQ, such as the ADOS. Have participants without a diagnosis but with high autistic traits been excluded from the non-ASC group (because they may have undiagnosed ASC and could be a confound)?

The authors are grateful for pointing out this shortcoming in the description of the group. All subjects in the clinical group had a formal diagnosis of autism spectrum disorder, which was additionally confirmed by the result of the AQ questionnaire. The description of the group has been changed in the new version of the text.

39. Inconsistent: the participant group is referred to as ASC as well as ASD. If the group doesn’t actually consist of individuals with ASC, it shouldn’t be referred to as such. High autistic traits vs. low autistic traits would be more appropriate than ASC vs control.

As mentioned in the previous item, the clinical group comprised entirely people diagnosed with autism spectrum disorder. The nomenclature throughout the text has been harmonized.

40. Explain the cut off point of the AQ.

The AQ questionnaire has additional information in its description that the results of 31 points and above suggest an ASD diagnosis. The studies have been carried out on the Polish population. Individuals with a 31+ score meet ASD criteria according to DSM IVTR (Baron-Cohen et al., 2001); Polish version Pisula (2010).

41. It is not made clear which of the questionnaires measures interoception, and what type of interoceptive processing (accuracy, sensitivity, sensibility).

The description of the BPQ Body Perception Questionnaire - Short Version (Porges, 1993, 2015) has been modified. It is a self-report questionnaire that measures body awareness (interoceptive sensibility) and autonomic reactivity. The questions in the questionnaire have been developed based on the structure of autonomous nervous system (ANS), the afferent signals of which carry information about the condition of organs and tissues, thus constituting the basis for the subjective awareness of the individual’s body (BPQ, BPQ-SF, BPQ-VSF).

42. From the description of the questionnaires, it appears that there is some overlap between the DERS and the TAS-20. How are they different? Do they correlate? How meaningful are the findings if there is multicollinearity? In the mediation model, when assessing the relationship between the TAS-20 and somatoform dissociation, has the DERS been controlled for? And similarly, when assessing the relationship between DERS and somatoform dissociation, has TAS-20 been controlled for?

In the new statistical model that combines DERS and TAS-20, a non-standard macro for SPSS PROCESS was used, and due to the use of Model 6, which enables the construction of a serial-multiple mediation model, the mutual relationship between DERS and TAS was controlled, as Model 6 assumes that the mediators will be strongly correlated, and so it was in this case.

Results

Page 14

43. “A single factor analysis of variance was carried out in an intergroup schema” is a strange way to describe the analysis done. Please make clearer what the independent and dependent variables are. With multiple dependent variables, isn’t a manova more appropriate? It doesn’t seem any post-hoc analyses were done after the (m)anova. In the table, the post-hoc statistics should be reported.

The analysis of variance was performed only to compare the clinical group with the control group in terms of other variables such as alexithymia, emotion dysregulation or interoception. In the new manuscript, this method has been changed to a t-test for independent samples, and *d-cohen* has been added to it.

44. In the description of the correlations, it is not always made clear which variables were correlated. Most of the time only one variable is mentioned, and it is unclear what other variable the correlation refers to.

The main assumption for presenting linear relationships was to show the relationship between somatoform disorders and alexithymia, emotion dysregulation and interoception, hence the main emphasis on the description of results in this respect.

Page 15

45. It is unclear why the authors have chosen to do a moderation analysis with high/low AQ as the moderator (as suggested by the graph) and not the two participant groups. In the graph, what does AQ yes AQ no mean? Surely all participants have an AQ result? Please use a more appropriate description. The description of the moderation analysis results is grammatically poorly written and is repetitive. The one result is described multiple times as if each description is a new finding.

The nomenclature throughout the text has been harmonized, which in this case has been changed according to the group description to the clinical group and the control group.

In addition, the description of moderation analysis results has been modified.

Page 16

46. It is unclear how the ‘obtained results’ of the moderation analysis ‘form the basis’ for the mediation analyses. Also, that authors state that it confirms the relationship in the ‘examined group’ (singular), not groups (plural). This is confusing, as it is unclear which group the authors refer to, and also, the moderation was confirmed for both groups.

The entire data analysis part has been modified. A new order has been given. It also presents the sequence of the analyses carried out and the results before their description, which is intended to ensure the logic of the performed statistical analyses.

47. Please don’t use the word ‘proven’

The authors are grateful for the suggestion.

Page 17

48. Here the authors specifically mention that the analysis shows (not: ‘achieved’) a partial mediation. This was however also the case with the two earlier mediation analyses.

The entire Results section has been modified in order to present the results more clearly.

49. In the summary of the mediation analyses results, the authors mention only the ASC group. Were the mediation analyses carried out on data from the ASC group only, not all participants? This is unclear, and if so, why? Why haven’t the authors carried out a moderated mediation analysis, which is possible in PROCESS? This would show the effect of the moderation of autism status on the mediated relationship between interoception and somatoform dissociation.

I hope that the revised part of Data Analysis and Results will resolve any doubts.

The statistical analysis was performed in certain steps, each of which resulted from the previous one. In a situation where a significant moderation effect was obtained, which shows that the dependence in the clinical group is the same as in the control group, and the only difference is the strength of this effect. A decision was made to perform further analyses on the entire group. The new paragraph on data analysis provides more consistency before the reader moves on to the Results section and this is described therein. It is worth noting that the last model has been changed from the parallel mediation to the serial multiple mediation model, which makes all the difference when it comes to adding a moderator to the model, because it would make the model much more difficult to perceive.

Discussion

Page 18

50. Please state the results in terms of the research question and hypotheses – why and how were the RQ and hypotheses formulated – ‘to verify a model’ is not informative. Again, sentences are poorly constructed, which impedes clarity. Please be consistent in the use of acronyms (what is DIF? Why do the authors write out somatoform dissociation in full, but use an acronym for SA, which wasn’t measured in the study, and the reader may have forgotten what it stands for.

The discussion of the results has been changed due to other statistical analyses.

51. The authors should make clear when they are speculating about mechanisms that may explain the findings, such as SA. Some statements are too strong and suggest causality when they can’t be sure of that.

Some of issues have been moved from the Discussion to the Introduction, including the one concerning somatosensory amplification.

52. The Discussion suffers from the same problem as the introduction: a list of descriptions of previous research, of which the relevance (to the authors’ findings this time – in the introduction it’s the relevance to the hypotheses and research question) is not made clear; supporting and opposing research findings are not clearly pointed out (everything is treated as supporting, even when it’s not); definitions are not clear – somatoform dissociation is equated with physical problems, physical illness, somatic amplification etc. It doesn’t address the question why these findings are worth knowing – how will this research help people with autism or what is its contribution to knowledge specifically?

The comments/remarks from the Review have been incorporated into the new structure of the entire article, including the discussion of results, conclusions, and limitations.
