# Supplementary material for: Relationship between interoceptive sensibility and somatoform disorders in adults with autism spectrum traits. The mediating role of alexithymia and emotional dysregulation
Source: PLoS One. 2021 Aug 24;16(8):e0255460. doi: 10.1371/journal.pone.0255460 (PMC8384168; doi:10.1371/journal.pone.0255460)
Supplement: Attachment — Submitted filename: Review Plos manuscript.docx [file pone.0255460.s004.docx]

Review of revised manuscript.

I’d like to congratulate the authors on a much clearer, better written manuscript.

Therefore my suggestions to improve further on the article consists mostly of only minor recommendations.

Introduction

1. Page 3, 1st paragraph: “Numerous studies focus on the fact…” – please cite a few of these numerous studies here.
2. Page 3, 2nd paragraph: “Based on the study results…” Do the authors mean their own study? It is unusual to announce one’s own results in the introduction, especially as a basis for anything (this is circular reasoning). A hypothesis should be presented here instead of results. If it is not the authors’ study, please cite the study this remark refers to.
3. Page 5, 2^nd^ paragraph: “An individual […] may cope using invasive defense mechanisms”. What are invasive defense mechanisms? I suspect the word invasive is not used correctly here. Do the authors mean maladaptive defense mechanisms?
4. Page 5, 2^nd^ paragraph: “Such a role of alexithymia…” Alexithymia is a condition, a personality trait. Are the authors suggesting that alexithymia is an example of an ‘invasive defense mechanism’? I am not sure this is correct, nor what Porcelli and Taylor intended to emphasize about alexithymia. Yes, alexithymia may compound maladaptive behaviours, but it is not a behaviour (so not a defense mechanism) itself. Please clarify, probably in conjunction with the sentence before (my point 3).
5. Page 6, 3^rd^ paragraph: Spelling/typo mistake: emptions instead of emotions
6. Page 6, 3^rd^ paragraph: There is no diagnosis for interoception disorders. There’s not even an official condition/disorder with regards to interoception, except perhaps the rare neurological condition of congenital analgesia, which is something entirely different. Altered interoception has been found in individuals with ASD; they weren’t diagnosed with an interoception disorder. Please remove the words ‘diagnosis’ and ‘disorder’ when referring to interoception, and rephrase.
7. Page 7, 1^st^ paragraph: the sentence “… that individuals with ASD suffer from numerous disorders of etiology with is difficult to determine” is grammatically incorrect. There’s no such thing as ‘disorders of etiology’, which is what the sentence suggests in its current form. Rephrase.
8. Page 9-10: The attachment disorder explanation of emotional difficulties in ASD is not a generally accepted explanation of the emotional deficits in ASD. It is therefore surprising that the authors cite a study (Minnis et al) which shows that only 2% of children with ASD have an attachment disorder as evidence for this claim. It is more logical to cite this as evidence of the weakness of this explanation, given the low prevalence of attachment disorder. Similarly, the authors cite Leonardi as evidence for alexithymia in parents of children with ASD to support this attachment explanation, when in fact differences in the levels of alexithymia found in parents with ASD children compared to parents with nonASD children were only found with a structured interview but not with the TAS20 questionnaire, AND the levels found were well below the threshold of alexithymia (so not ‘typical for parents’ as the authors claim). This is not strong evidence at all, and certainly not for what the authors seem to be claiming; that alexithymia in parents results in poor attachment with ASD children. The authors then cite a number of other studies showing reduced emotion recognition, reduced focus on faces etc, as evidence for this attachment disorder explanation, when these studies do not provide evidence for the attachment approach at all. Especially the 1^st^ paragraph of page 10 “A logical approach [….] which shows that these deficits do not constitute a key feature of this disorder” is incoherently and far too strongly phrased (The Uljarevic meta-analysis did not investigate attachment at all). I would suggest the authors just describe the emotional deficits shown in autism and only briefly (if at all) refer to attachment disorder, not as an explanation but as a factor that may contribute, for a small minority of individuals with ASD.
9. Page 11, final paragraph: “A significant amount of data indicates a strong relationship between alexithymia and interoception”. Remove the word ‘strong’. A strong relationship suggests a correlation of 0.7 or higher; this has not been found between alexithymia and interoception.
10. Page 11, final paragraph: “Craig suggests an exceptionally broad approach…” Remove the word ‘exceptionally’. Craig’s definition/description of interoception is generally accepted and is not exceptional at all.
11. Starting page 12, bottom, until page 13, the bit about Pellicano and Burr. I suggest that the description of Rogers and Ozonoff’s work and hyper/hyposensitivity in ASD is taken out. This refers to exteroception, not interoception, and is confusing. The relationship with predictive coding in the rest of the paragraph is also unclear.
12. Page 13, first paragraph, last sentence. The work of Mul et al (2018) and Shah et al (2016) and Cook et al (2013) needs to be cited here too. Please note that all studies have been correlational. It could well be that reduced interoception is responsible for alexithymia, it is not necessarily the other way around. I suggest to rephrase ‘could well be responsible’.
13. Page 14, first paragraph: “As a result, persons who have difficulty identifying feelings may also have less access…”. Remove the words ‘as a result’. Craig suggests that feelings and emotions ARE (interpreted) signals coming from the body. As I indicated earlier, causation is not clear and should therefore not be presumed. People with alexithymia may be less good at interpreting emotions/feelings, because they may not feel the signals as strongly. See also Brewer et al (2016), which the authors cite themselves.
14. Page 17, first paragraph: Finish the introduction with a recap of what the study investigates. Just one or two sentences will do.

Method

No suggestions. Very clear now.

Results:

1. Please use the correct descriptions for the strength of correlations found. ‘high’ and ‘strong’ are not used correctly here. The correlations found are in the weak to moderate range.
2. Page 24, first paragraph. “… the subsequent analyzes of mediations….”. Typo/Spelling error: should be ‘analyses’
3. Please be consistent. Sometimes the authors refer to ‘emotion regulation’ and at other times to ‘emotion dysregulation’. Choose one name for the variable.
4. In the captions explaining the graphs, (which are very clear, my congratulations), please mention the questionnaires the variables that are depicted were measured with.

Discussion

1. Page 32-33, first paragraph and throughout. Please take out all the causal language, e.g. ‘causes’, ‘leading to’, ‘as a consequence’, ‘as a result’, ‘hence’, and substitute with less strong words like ‘contributes to’, ‘is associated with’. As the direct effect remained significant in all mediation analyses, alexithymia and emotion regulation have been found to be partial mediators, not full mediators, and the role of emotion regulation disappeared entirely when alexithymia was taken into account. Strong words are not appropriate.
2. I think the authors should question openly whether emotion regulation can actually be considered a mediator, instead of stating confidently that their hypothesis re. emotion regulation was confirmed. Especially since the subscales that showed a mediating role were ones that seem to be measuring symptoms that are very similar to alexithymia (lack of emotional awareness and lack of emotional clarity). It seems to me that alexithymia was measured by these subscales, and not so much emotion regulation. This would explain why the mediating effect disappears when alexithymia is already taken into account in the simultaneous model. More alexithymia does not significantly add anything. Nor do the other subscales that measure actual emotion regulation, clearly, as they weren’t mediators in the first place.
3. Page 35, 1^st^ paragraph “Our findings are key as they resolve literature inconsistencies relating to social and emotional processes….”. The authors overstate and overinterpret their findings here, because they haven’t measured any social processes, and several other papers have already addressed the role of alexithymia vs the role of ASD in these processes, which the authors even cite. Please remove or tone down considerably.
4. Page 35, 2^nd^ paragraph, last sentence: Although the authors use the correct term ‘contributes to’ here, they confuse the reader with stating a direction of the relationship with interoceptive sensibility. Their models and most of the literature they have cited propose that interoception contributes to alexithymia.

Conclusion

1. In the Conclusion (and in the final paragraph of the Discussion) the authors should reflect on point 2 above (Discussion).
