# Supplementary material for: Relationship between interoceptive sensibility and somatoform disorders in adults with autism spectrum traits. The mediating role of alexithymia and emotional dysregulation
Source: PLoS One. 2021 Aug 24;16(8):e0255460. doi: 10.1371/journal.pone.0255460 (PMC8384168; doi:10.1371/journal.pone.0255460)
Supplement: Attachment — Submitted filename: response to Reviewers0205.docx [file pone.0255460.s005.docx]

I’d like to congratulate the authors on a much clearer, better written manuscript.

Therefore my suggestions to improve further on the article consists mostly of only minor recommendations.

Thank you very much for your comments. We have considered carefully all your advice, which we appreciate very much. As suggested by the Reviewer, we have reconstructed the manuscript. We hope its structure is clearer. All comments listed below have been incorporated into the modified version.

Abstract:

This statement below needs to be reworded as it seems to be based on speculations (and causation) yet big claims. More to it, previous research investigating somatoform disorders and interoception (Schaefer et al., 2012) has not been mentioned in this study in which there was no evidence of increased interoception in somatoform disorders, as measured by cardiac perception tasks. I acknowledge that the variation in findings when using objective tasks vs self-reported measures. Nonetheless, the findings need to be interpreted with caution (I would suggest the same comment for your conclusion).
“It has been assumed that the interoceptive sensibility is accompanied by a high level of alexithymia and emotion dysregulation, which fosters somatosensory amplification and, as a consequence, results in the intensification of somatoform disorders”
Please avoid the word *proved to be significant*.
Thank you very much for your comment . The abstract has been modified as suggested.

Introduction

This introduction is very long and needs serious amendments (especially, when you consider in comparison to the discussion of findings, which generally needs more elaboration). I admire the authors efforts of providing comprehensive background but this is not a review hence, please make it concise. Right now, it is very hard to follow this introduction. I will expand my comment below:
Your current structure is:
• Interoceptive sensibility and somatoform disorders
• Alexithymia and emotion dysregulation
• Interoception, alexithymia and somatosensory amplification
• Somatoform symptoms (positive and negative) (this and all of the above need to be made concise and in order; you might benefit from subheadings)
• Autism spectrum disorder (ASD); Hypothesis and aims; ASD (All of these need to be re-ordered; hypothesis and rationale should be at the end)
• Interoception and alexithymia in autism; Emotional dysregulation (starting with general interoception, which should be much earlier)
• Neuronal correlates of alexithymia
• Alexithymia and self-reported interoception (this section and the above, neuronal correlates can be made much concise in a few lines)
• Dissociation, alexithymia - regulation of emotions and somatization (contrary to the above, this is an important part of the study; hence the detail makes sense)
• Following this, you again touch upon alexithymia and emotions (deficits in the cognitive processing). This section was already covered in the beginning. The repetition does not make sense or let’s say the order i.e., why would you mention it just before the Method section while it should have been the hypotheses or the study rationale here.
I would suggest you to make subheadings in the following order:
1. Somatoform disorders (including symptoms)
2. Interoceptive sensibility and somatoform disorders (all interoception general details like types or measurement should be covered here too)
3. Aims of the study
4. Autism spectrum disorder and the relevant details
5. Interoception, alexithymia, emotion dysregulation, ASD and dissociation
5.1. Alexithymia and emotion dysregulation
5.2. Interoception and alexithymia (here you can mention neural correlates too, briefly)
5.3. Interoception, alexithymia and emotions in ASD
5.4. Dissociation, alexithymia, emotions and somatization (ASD too if possible)
6. Hypothesis and study rationale
I would re-emphasise that you use sub-headings as this is a lot of information so, try to keep it concise.
Thank you very much for the suggestion to edit the theoretical part of the manuscript. According to the proposed plan and structure, changes were introduced. We hope that the logic of the argument is much clearer now.

1. Page 3, 1st paragraph: “Numerous studies focus on the fact…” – please cite a few of these numerous studies here.

Thank you very much. This passage has been completely changed.

2. Page 3, 2nd paragraph: “Based on the study results…” Do the authors mean their own study? It is unusual to announce one’s own results in the introduction, especially as a basis for anything (this is circular reasoning). A hypothesis should be presented here instead of results. If it is not the authors’ study, please cite the study this remark refers to.

It was a misunderstanding. This passage has been completely changed.

3. Page 5, 2^nd^ paragraph: “An individual […] may cope using invasive defense mechanisms”. What are invasive defense mechanisms? I suspect the word invasive is not used correctly here. Do the authors mean maladaptive defense mechanisms?

Thank you very much for your correction. We have tried to write the text properly, but it seems that we have made a mistake. We have changed the sentence.

Page 5, 2^nd^ paragraph: “Such a role of alexithymia…” Alexithymia is a condition, a personality trait. Are the authors suggesting that alexithymia is an example of an ‘invasive defense mechanism’? I am not sure this is correct, nor what Porcelli and Taylor intended to emphasize about alexithymia. Yes, alexithymia may compound maladaptive behaviours, but it is not a behaviour (so not a defense mechanism) itself. Please clarify, probably in conjunction with the sentence before (my point 3).

Thank you very much for your correction. We have tried to write the text properly, but it seems that we have made a mistake. We have changed the sentence

4. Page 6, 3^rd^ paragraph: Spelling/typo mistake: emptions instead of emotions

Thank you for correction.

5. Page 6, 3^rd^ paragraph: There is no diagnosis for interoception disorders. There’s not even an official condition/disorder with regards to interoception, except perhaps the rare neurological condition of congenital analgesia, which is something entirely different. Altered interoception has been found in individuals with ASD; they weren’t diagnosed with an interoception disorder. Please remove the words ‘diagnosis’ and ‘disorder’ when referring to interoception, and rephrase.

Thank you very much for your comment. We have made appropriate changes to the terminology in the manuscript.

6. Page 7, 1^st^ paragraph: the sentence “… that individuals with ASD suffer from numerous disorders of etiology with is difficult to determine” is grammatically incorrect. There’s no such thing as ‘disorders of etiology’, which is what the sentence suggests in its current form. Rephrase.

Thank you for correction.

7. Page 9-10: The attachment disorder explanation of emotional difficulties in ASD is not a generally accepted explanation of the emotional deficits in ASD. It is therefore surprising that the authors cite a study (Minnis et al) which shows that only 2% of children with ASD have an attachment disorder as evidence for this claim. It is more logical to cite this as evidence of the weakness of this explanation, given the low prevalence of attachment disorder. Similarly, the authors cite Leonardi as evidence for alexithymia in parents of children with ASD to support this attachment explanation, when in fact differences in the levels of alexithymia found in parents with ASD children compared to parents with nonASD children were only found with a structured interview but not with the TAS20 questionnaire, AND the levels found were well below the threshold of alexithymia (so not ‘typical for parents’ as the authors claim). This is not strong evidence at all, and certainly not for what the authors seem to be claiming; that alexithymia in parents results in poor attachment with ASD children. The authors then cite a number of other studies showing reduced emotion recognition, reduced focus on faces etc, as evidence for this attachment disorder explanation, when these studies do not provide evidence for the attachment approach at all. Especially the 1^st^ paragraph of page 10 “A logical approach [….] which shows that these deficits do not constitute a key feature of this disorder” is incoherently and far too strongly phrased (The Uljarevic meta-analysis did not investigate attachment at all). I would suggest the authors just describe the emotional deficits shown in autism and only briefly (if at all) refer to attachment disorder, not as an explanation but as a factor that may contribute, for a small minority of individuals with ASD.

In this section, we have made significant abbreviations in line with the remarks. Thank you for your comment.

8. Page 11, final paragraph: “A significant amount of data indicates a strong relationship between alexithymia and interoception”. Remove the word ‘strong’. A strong relationship suggests a correlation of 0.7 or higher; this has not been found between alexithymia and interoception.

Thank you very much for your correction. Changes have been made throughout the manuscript.

9. Page 11, final paragraph: “Craig suggests an exceptionally broad approach…” Remove the word ‘exceptionally’. Craig’s definition/description of interoception is generally accepted and is not exceptional at all.

Thank you. The change has been made.

10. Starting page 12, bottom, until page 13, the bit about Pellicano and Burr. I suggest that the description of Rogers and Ozonoff’s work and hyper/hyposensitivity in ASD is taken out. This refers to exteroception, not interoception, and is confusing. The relationship with predictive coding in the rest of the paragraph is also unclear.

As suggested, this section has been removed from the manuscript.

11. Page 13, first paragraph, last sentence. The work of Mul et al (2018) and Shah et al (2016) and Cook et al (2013) needs to be cited here too. Please note that all studies have been correlational. It could well be that reduced interoception is responsible for alexithymia, it is not necessarily the other way around. I suggest to rephrase ‘could well be responsible’.

The citations were introduced.

12. Page 14, first paragraph: “As a result, persons who have difficulty identifying feelings may also have less access…”. Remove the words ‘as a result’. Craig suggests that feelings and emotions ARE (interpreted) signals coming from the body. As I indicated earlier, causation is not clear and should therefore not be presumed. People with alexithymia may be less good at interpreting emotions/feelings, because they may not feel the signals as strongly. See also Brewer et al (2016), which the authors cite themselves.

Thank you very much for your correction. We have tried to write the text properly, but it seems that we have made a mistake.

13. Page 17, first paragraph: Finish the introduction with a recap of what the study investigates. Just one or two sentences will do.

Method

No suggestions. Very clear now.

Procedure:
Was the order of completing the questionnaires same or randomised? Please mention

Thank you very much for your comment. We have added this information.

The subheadings with each questionnaire, how its measured and psychometric properties make the section easy to follow.
BPQ: Since you explain autonomic reactivity well, it would be nice to see the same for body perception questionnaire. You might want to add an example or item of these to illustrate especially, for novice readers.
Sample BPQ questions have been added to the manuscript.

Results:

1. Please use the correct descriptions for the strength of correlations found. ‘high’ and ‘strong’ are not used correctly here. The correlations found are in the weak to moderate range.

Thank you very much for your correction.

2. Page 24, first paragraph. “… the subsequent analyzes of mediations….”. Typo/Spelling error: should be ‘analyses’

Thank you very much for your correction.

3. Please be consistent. Sometimes the authors refer to ‘emotion regulation’ and at other times to ‘emotion dysregulation’. Choose one name for the variable.

Thank you very much for your correction.

4. In the captions explaining the graphs, (which are very clear, my congratulations), please mention the questionnaires the variables that are depicted were measured with.

Thank you very much for your correction. Adjustments were made as suggested.

5. Typo: initial analyzes – I assume this is initial analyses. Please correct that. (Same for *more advanced statistical analyzes* just before it says insert figure 2 and 3 here)
Thank you for correction.

“which confirm the direct impact” It might be better to use “support” instead of confirm

Thank you for correction.

Discussion

1. Page 32-33, first paragraph and throughout. Please take out all the causal language, e.g. ‘causes’, ‘leading to’, ‘as a consequence’, ‘as a result’, ‘hence’, and substitute with less strong words like ‘contributes to’, ‘is associated with’. As the direct effect remained significant in all mediation analyses, alexithymia and emotion regulation have been found to be partial mediators, not full mediators, and the role of emotion regulation disappeared entirely when alexithymia was taken into account. Strong words are not appropriate.

Thank you very much for your correction. We've made some adjustments throughout this manuscript.

2. I think the authors should question openly whether emotion regulation can actually be considered a mediator, instead of stating confidently that their hypothesis re. emotion regulation was confirmed. Especially since the subscales that showed a mediating role were ones that seem to be measuring symptoms that are very similar to alexithymia (lack of emotional awareness and lack of emotional clarity). It seems to me that alexithymia was measured by these subscales, and not so much emotion regulation. This would explain why the mediating effect disappears when alexithymia is already taken into account in the simultaneous model. More alexithymia does not significantly add anything. Nor do the other subscales that measure actual emotion regulation, clearly, as they weren’t mediators in the first place.

Thank you very much for your comments. A part concerning the role of alexithymia in the context of dysregulation was added to the discussion.

3. Page 35, 1^st^ paragraph “Our findings are key as they resolve literature inconsistencies relating to social and emotional processes….”. The authors overstate and overinterpret their findings here, because they haven’t measured any social processes, and several other papers have already addressed the role of alexithymia vs the role of ASD in these processes, which the authors even cite. Please remove or tone down considerably.

Thank you very much for your correction. We have tried to write the text properly, but it seems that we have made a mistake.

4. Page 35, 2^nd^ paragraph, last sentence: Although the authors use the correct term ‘contributes to’ here, they confuse the reader with stating a direction of the relationship with interoceptive sensibility. Their models and most of the literature they have cited propose that interoception contributes to alexithymia.

Thank you very much for your comment. We have changed the sentence.

When you mention hypothesis, the word “supported” is better than confirmed.
“greater intensity of both interoceptive sensitivity”. I assume you mean interoceptive sensibility. The same comment reflects “increased sensitivity”. Considering interoception literature, these terms can cause confusion. Please use the appropriate terms.
“deeper somatoform symptoms” what do you mean by deeper?
“proved a strong relationship” Please avoid the word prove/d
Thank you very much for your correction. All changes have been made throughout the text

“These physical illnesses are the consequence of individual suffering from alexithymia having an inability to appreciate, distinguish, and express affect, which in turn, increases the physiological arousal and those negative subjective states that are not governed by psychological strategies”.
Which physical illnesses? Do you mean in general, any named or more so, in terms of somatoform disorders? This seems a claim and needs a reference.
Thank you very much for your correction. Physical illnesses changed to somatoform disorders.

There is too much focus on alexithymia and emotions in autism, which is already quite a common knowledge given the enormous amount of literature available. This diverges the discussion focus from the core area e.g., what are the central elements of this research or in other words, implications? Any possible explanation on the difference found on the autonomic reactivity scale only and not the body perception? Discussion of the findings in relation to the previous research on somatoform disorders and interoception? What next?
Figure 2 (and all other relevant fields related to it): It implies that excessive interoception causes somatoform disorders. What if it’s the other way around? In other words, correlation is not causation. Please review this throughout the manuscript including the results where you mention “direct”
Thank you very much for your correction. We have tried to write the text properly, but it seems that we have made a mistake.
Grammar – please proof read the manuscript. There are several grammar errors, which makes the reading hard.
Thank you very much for your comments.

Overall, throughout the manuscript, there are some strong statements implying causal mechanisms. These should be reworded.
Nonetheless, authors’ efforts are much appreciated.

Conclusion

1. In the Conclusion (and in the final paragraph of the Discussion) the authors should reflect on point 2 above (Discussion).

Thank you very much for your comment. In the discussion of the results, we discussed the significant role of alexithymia in the relationship between interoceptive sensibility and somatoform disorders, and the lack of a significant role of emotional dysregulation in this model.

Thank you very much for your all your recommendations. We have tried to revise all the manuscript taking into account all your comments. We hope you find it suitable.
