# Supplementary material for: Relationship between interoceptive sensibility and somatoform disorders in adults with autism spectrum traits. The mediating role of alexithymia and emotional dysregulation
Source: PLoS One. 2021 Aug 24;16(8):e0255460. doi: 10.1371/journal.pone.0255460 (PMC8384168; doi:10.1371/journal.pone.0255460)
Supplement: Attachment — Submitted filename: Response to Reviewers.docx [file pone.0255460.s006.docx]

Response to Reviewers

Dear Reviewers,

Thank you so much for your time, attention, patience and extensive knowledge. Thank you very much for your all your recommendations.

*Review of revised manuscript.*

*Reviewer #1: May I congratulate the authors with a much improved and much stronger, very interesting paper. Well done!*

*Reviewer #2: Well done to authors for improving the manuscript massively. I suggest a few minor changes below*

Thank you very much for your acceptance and recognition of our substantive contribution as enough.

Below are comments on minor changes that appeared in Reviewer 2 report

*Introduction*

*“Interoception refers to the ability to accurately perceive internal bodily processes ….” Instead of “IA is defined as the ability …”*

*Therefore, please replace “IA” with “interoception” in the context where it applies (especially given that you are measuring interoceptive sensibility in your study).*

Thank you very much for your correction. We have changed the sentence.

*In the section, “hypothesis and study rationale”, please consider starting with: “It was hypothesized that” paragraph*

Thank you so much. The clarity of arguments on this change has significantly improved.

*Discussion: i.e., in paragraph 1 In our study, (comma after study)*

Thank you. The comma has been made.
